# Host susceptibility spectrum and diagnostic challenges in pediatric nontuberculous mycobacterial disease: a case series of five patients with literature review

**DOI:** 10.3389/fped.2026.1852871

**Published:** 2026-06-19

**Authors:** Yan Ruan, Muxing Chen, Jiandong Lin, Huiyun Huang, Fei Lin, Weiwei Tang

**Affiliations:** 1 Department of Pediatrics and Central Laboratory, Fujian Medical University Clinical Teaching Hospital, Fuzhou Pulmonary Hospital of Fujian Province, Fuzhou, Fujian, China; 2 Department of Tuberculosis and Central Laboratory, Fujian Medical University Clinical Teaching Hospital, Fuzhou Pulmonary Hospital of Fujian Province, Fuzhou, Fujian, China

**Keywords:** bronchiectasis, children, cystic fibrosis, *Mycobacterium abscessus*, *Mycobacterium paragordonae*, nontuberculous mycobacteria

## Abstract

**Objective:**

Pediatric nontuberculous mycobacterial (NTM) infections are frequently misdiagnosed due to their heterogeneous clinical presentations, which can mimic pulmonary tuberculosis, common respiratory diseases, or other infectious conditions depending on the site of involvement. This case series aimed to characterize the host susceptibility spectrum underlying pediatric NTM infection across both pulmonary and extrapulmonary sites, with emphasis on the diagnostic role of targeted next-generation sequencing (tNGS).

**Methods:**

We conducted a retrospective analysis of five pediatric patients with confirmed NTM infection diagnosed at Fujian Provincial Fuzhou Pulmonary Hospital between January and December 2025. Clinical characteristics, predisposing factors, therapeutic regimens, and clinical outcomes were systematically reviewed and discussed in the context of relevant literature.

**Results:**

The cohort included 5 children aged 7 months to 12 years. Pathogenic species were *Mycobacterium abscessus* (*n* = 4) and *Mycobacterium paragordonae* (*n* = 1). Infection sites included the lungs (*n* = 3), cervical lymph nodes (*n* = 1), and parotid gland (*n* = 1). Predisposing factors ranged from localized infection in immunocompetent children to congenital airway anomalies, DiGeorge syndrome, and cystic fibrosis with bronchiectasis. All patients improved after individualized multidrug therapy and were discharged.

**Conclusions:**

Pediatric NTM infection is associated with a broad spectrum of predisposing factors, including bronchiectasis, cystic fibrosis, congenital airway anomalies, and systemic immunodeficiency such as DiGeorge syndrome. tNGS demonstrated significant diagnostic value in identifying NTM species and should be considered when conventional methods are insufficient.

## Introduction

Nontuberculous mycobacteria (NTM) are ubiquitously distributed in natural water sources, soil, aerosols, and medical water systems (1). NTM infections are generally acquired through environmental exposure and were traditionally considered incapable of person-to-person transmission; however, recent whole-genome sequencing studies have suggested that *Mycobacterium abscessus* may undergo inter-human transmission in specific patient populations (2, 3).

Globally, both the incidence and detection rates of NTM infections have demonstrated a sustained upward trajectory. The annual incidence of pediatric NTM infection varies considerably across countries and regions, ranging from 0.6 to 5.36 per 100,000 children, with the highest reported values observed in Europe, where the trend continues to rise (4). In the United States, the pediatric NTM-related hospitalization rate increased from 1.2 per 10,000 discharges in 2000 to 2.6 per 10,000 discharges in 2022, representing a more than twofold increase over a 22-year period (5). In mainland China, the NTM isolation rate among patients with suspected tuberculosis is 6.3% (95% CI: 5.4%–7.4%), with the highest NTM prevalence reported in the southeastern regions; *Mycobacterium abscessus* and *Mycobacterium avium* complex constitute the predominant pathogenic species (6). Due to its intrinsic resistance to the vast majority of conventional antimicrobial agents, *M. abscessus* has emerged as one of the most therapeutically challenging NTM species in the pediatric population (7, 8). Concurrently, with the expanding clinical adoption of targeted next-generation sequencing (tNGS), certain rare NTM species — such as *Mycobacterium paragordonae* (9) — have been identified in pediatric cases, although such reports remain exceptionally uncommon.

Herein, we retrospectively report five pediatric cases of NTM infection managed at Fujian Provincial Fuzhou Pulmonary Hospital in 2025. The pathogenic species included *M. abscessus* (*n* = 4) and *M. paragordonae* (*n* = 1), with sites of infection encompassing the lungs, cervical lymph nodes, and parotid gland. The cases collectively illustrate a diverse range of host susceptibility conditions that facilitate NTM infection in children — from disruption of physical barriers and impaired mechanical airway defense to systemic cellular immunodeficiency. Through detailed case analysis and an integrative literature review, this report aims to enhance clinical recognition of predisposing factors for pediatric NTM infection, and to elucidate the practical diagnostic value of tNGS in the identification of rare mycobacterial species and the evaluation of complex infectious presentations.

## Case presentation

All five pediatric patients were treated at Fujian Provincial Fuzhou Pulmonary Hospital from January to December 2025. Etiological diagnoses were confirmed by tNGS, fluorescence PCR melting curve analysis, and/or histopathological examination. The diagnostic criteria for NTM disease were applied in accordance with the Guidelines for the Diagnosis and Treatment of Nontuberculous Mycobacterial Disease (2020 Edition), issued by the Tuberculosis Branch of the Chinese Medical Association (10). A summary of the clinical data for all five patients is presented in Table 1.

**Table 1:** Clinical characteristics of five pediatric patients with NTM infection.

Case	Sex	Age	Site of Infection	Pathogenic Species	Potential Predis-posing Factors	Diagnostic Method	Treatment Regimen	Outcome
1	Male	9 yr	Parotid gland	*M. paragordonae*	localized infection	Tissue tNGS	INH + RFP + EMB + CLR	Improved
2	Female	10 yr	Cervical lymph nodes	*M. abscessus*	localized infection	Histopathology + fluorescence PCR melting curve analysis of discharge specimen	Local AMK irrigation + CFX + AMK + AZM + LZD	Improved
3	Female	12 yr	Lung	*M. abscessus*	Cystic fibrosis; bronchiectasis	Sputum/BALF tNGS + positive acid-fast staining	CLR + LZD + LFX	Improved
4	Male	7 mo	Lung	*M. abscessus*	Congenital laryngomalacia; bronchomalacia	BALF tNGS + fluorescence PCR melting curve analysis	AZM + CFX + IMP → AZM + CFX + LZD	Improved
5	Male	6 mo	Lung	*M. abscessus*	DiGeorge syndrome; congenital laryngomalacia; bronchomalacia	BALF tNGS	AZM + CFX + AMK + LZD + T*α*1	Improved

NTM, nontuberculous mycobacteria; *M. abscessus*, *Mycobacterium abscessus*; *M. paragordonae*, *Mycobacterium paragordonae*; tNGS, targeted next-generation sequencing; BALF, bronchoalveolar lavage fluid; AZM, azithromycin; LFX, levofloxacin; CFX, cefoxitin; IMP, imipenem-cilastatin; LZD, linezolid; AMK, amikacin; INH, isoniazid; RFP, rifampicin; EMB, ethambutol; CLR, clarithromycin. Tα1, thymosin alpha-1. yr, year(s); mo, month(s).

tNGS was performed selectively based on clinical suspicion and the inadequacy of conventional diagnostic methods. Clinical specimens, including tissue biopsy, sputum, and bronchoalveolar lavage fluid (BALF), were processed for multiplex targeted amplicon-based high-throughput sequencing at certified clinical laboratories (accredited under CAP, ISO 15189, or equivalent standards). All assays employed a targeted amplification strategy covering predefined panels of respiratory pathogens and/or mycobacterial species with drug-resistance gene profiling, utilizing validated high-throughput sequencing platforms with automated bioinformatics analysis and integrated pathogen reference databases. Given the targeted amplification-based design of all assays, detection sensitivity was largely unaffected by host genomic background, as the targeted amplification step inherently minimizes interference from human genomic sequences. Positive pathogen identification was determined according to each laboratory's validated internal criteria; sequence read counts supporting each identification are reported in the respective case descriptions. All tNGS results were interpreted in conjunction with clinical presentation, imaging findings, and conventional microbiological or histopathological results to distinguish true infection from colonization or contamination.

### Case 1: *Mycobacterium paragordonae* infection of the parotid gland in an immunocompetent child

A 9-year-old boy with no significant past medical history was admitted with a chief complaint of “a right pre-auricular mass noted for more than 20 days.” Physical examination revealed a mass of approximately 1 cm × 1 cm with tenderness on palpation; the overlying skin showed no erythema or ulceration, and the patient was afebrile. Cervical ultrasonography demonstrated multiple enlarged right-sided cervical lymph nodes and a mixed-echogenicity parotid mass with calcification (approximately 2.65 cm × 0.65 cm). The patient underwent right parotid lesion excision and facial nerve decompression. Postoperative histopathology revealed chronic granulomatous inflammation with focal necrosis, and acid-fast staining was positive (+). These findings suggested that mycobacterial infection, including tuberculosis, could not be excluded. Tissue-based tNGS identified *Mycobacterium paragordonae* (117 sequence reads). T-/B-/NK-cell lymphocyte subset analysis was within normal limits. Chest computed tomography (CT) revealed no significant abnormalities.

The patient was ultimately diagnosed with *M. paragordonae* infection of the parotid gland. The patient was initiated on a regimen of isoniazid, rifampicin, ethambutol, and clarithromycin. Follow-up ultrasonography at 3 months postoperatively showed no recurrence of the right parotid lesion.

### Case 2: *Mycobacterium abscessus* infection of the cervical lymph node in an immunocompetent child

A 10-year-old girl with no significant past medical history was admitted with a chief complaint of “swelling and pain in the left submandibular and submental regions for more than two months.” Approximately two months prior to presentation, the patient first noticed left gingival pain, followed by a soft, non-erythematous mass of approximately 1 cm × 1 cm with associated tenderness and intact overlying skin without ulceration. The patient had been evaluated at multiple local clinics and hospitals, where the working diagnosis of acute lymphadenitis had been made; she received multiple courses of cephalosporin with partial symptomatic relief, but the mass progressively enlarged to approximately 2.5 cm × 2.5 cm upon discontinuation of antibiotics. She subsequently underwent exploratory surgery of the left cervicofacial mass at another institution. Intraoperatively, two soft masses of approximately 2.5 cm × 2.5 cm each, containing abundant caseous necrotic material, were identified. Histopathological examination demonstrated inflammatory lesions with necrosis, granuloma formation, and involvement of skeletal muscle. The diagnosis of *M. abscessus* infection was confirmed via fluorescence PCR melting curve analysis, which yielded consistently positive results across both the lymph node biopsy tissue and discharge specimens.

The patient was ultimately diagnosed with *M. abscessus* infection of the submental and left submandibular lymph nodes. Treatment consisted of local wound debridement with amikacin irrigation, combined with systemic therapy comprising cefoxitin, amikacin, azithromycin, and linezolid. At six-month follow-up, serial ultrasonography demonstrated significant reduction of the lesion.

### Case 3: *Mycobacterium abscessus* pulmonary disease in a child with cystic fibrosis

A 12-year-old girl was admitted with a chief complaint of “recurrent cough for 16 days.” She had a 10-year history of bronchial asthma, for which she had been irregularly inhaling budesonide/formoterol over the preceding year. Chest CT performed on admission revealed bilateral bronchial wall thickening with partial mild bronchiectasis, raising clinical suspicion for cystic fibrosis; the diagnosis was subsequently confirmed by detection of pathogenic *CFTR* gene mutations. Two independent tNGS analyses performed on sputum and bronchoalveolar lavage fluid (BALF) both identified *M. abscessus* (96 and 90 sequence reads, respectively), and BALF acid-fast staining was positive (2+).

The patient was ultimately diagnosed with *M. abscessus* pulmonary disease in the context of cystic fibrosis. The patient was treated with clarithromycin, levofloxacin, and linezolid for eight months, resulting in improvement of cough and sputum production. During the four-month follow-up period after treatment completion, multiple sputum mycobacterial cultures remained negative.

### Case 4: *Mycobacterium abscessus* pulmonary disease in an infant with congenital airway structural anomalies

A 7-month-old boy was admitted with a chief complaint of “cough and audible tracheal secretions for more than two weeks.” The infant had developed paroxysmal cough with noisy breathing approximately two weeks prior. Chest CT performed at a referring hospital had revealed multifocal patchy infiltrates in both lungs, prompting a diagnosis of severe pneumonia. An 8-day course of cefoperazone-sulbactam had resulted in symptomatic improvement, but follow-up chest radiography demonstrated inadequate resolution of pulmonary lesions. Subsequent bronchoscopic evaluation revealed mild laryngomalacia, severe bronchomalacia with slit-like linear stenosis of the right upper lobe and right lower lobe dorsal segmental bronchi, and bilateral endobronchial inflammation. BALF tNGS identified *M. abscessus* (16,474 sequence reads), and fluorescence PCR melting curve analysis of BALF confirmed *M. abscessus*.

The patient was ultimately diagnosed with *M. abscessus* pulmonary disease in the context of congenital laryngomalacia and bronchomalacia. Cefoperazone-sulbactam was discontinued, and the patient was initiated on azithromycin, cefoxitin, and imipenem-cilastatin. Following discharge, the regimen was modified to azithromycin, cefoxitin, and linezolid, resulting in clinical improvement and radiological regression of the pulmonary infiltrates over the three-month follow-up period.

### Case 5: *Mycobacterium abscessus* pulmonary disease in an infant with diGeorge syndrome

A 6-month-old boy was admitted with a chief complaint of “recurrent cough with fever for more than one month.” The infant presented with severe malnutrition and cachexia. His medical history was significant for congenital laryngomalacia, bronchomalacia, patent ductus arteriosus, patent foramen ovale, thymic hypoplasia, and an established diagnosis of DiGeorge syndrome. He had experienced recurrent cough and fever for over one month with inadequate response to anti-infective therapy. Chest CT demonstrated bilateral pneumonia with extensive pulmonary consolidation. BALF tNGS identified *M. abscessus* (821 sequence reads).

The patient was ultimately diagnosed with *M. abscessus* pulmonary disease in the context of DiGeorge syndrome. The patient was treated with a regimen of azithromycin, cefoxitin, amikacin, and linezolid, supplemented by intravenous immunoglobulin and thymosin alpha-1 (T*α*1). He became afebrile and demonstrated improvement in cough. Follow-up CT performed 3 months after initiation of treatment showed significant absorption of pulmonary lesions compared with prior imaging.

## Discussion

NTM are opportunistic pathogens whose pathogenicity is critically dependent on host immune status and the local microenvironment. The five pediatric cases presented in this series collectively illustrate the full spectrum of host predisposing conditions that facilitate NTM infection in children — from disruption of physical barriers (Cases 1 and 2) and impaired mechanical airway defense (Cases 3 and 4) to systemic cellular immunodeficiency (Case 5)— providing important insights into the multifactorial risk landscape of pediatric NTM disease. Furthermore, tNGS proved instrumental in this series, enabling accurate species-level identification of both the predominant Mycobacterium abscessus and the rare Mycobacterium paragordonae, which would likely have been missed or misidentified by conventional microbiological methods.

## Localized NTM infection in immunocompetent children

In immunocompetent individuals, NTM infections typically manifest as localized, tissue-invasive disease. Once the physical barrier of the skin or soft tissue is breached, NTM may gain entry and proliferate locally; this propensity is particularly pronounced for rapidly growing mycobacterial species such as *M. abscessus*, which demonstrates a predilection for relatively younger individuals (11). Cervicofacial lymphadenitis represents the most prevalent form of NTM infection in this population, and its pathogenesis is generally attributed to pathogen entry via micro-disruptions of the oral mucosa. Given the relative fragility of the oral mucosa in young children and their frequent environmental exposure to soil and water, this age group is particularly susceptible (12, 13). In a landmark 28-year multicenter cohort study, Martínez-Planas et al. described 311 microbiologically confirmed pediatric NTM cases, of which 308 (99%) involved the cervicofacial region. The median age at diagnosis was 2.4 years (IQR: 1.7–3.2 years), and the vast majority of patients were immunocompetent without any underlying comorbidities, suggesting that age-related immaturity of local immune function might be a critical predisposing factor for NTM cervical lymphadenitis in young children (13).

Despite these established patterns, NTM cervical lymphadenitis remains a diagnostic challenge due to its insidious onset and clinical features that substantially overlap with typical bacterial lymphadenitis. Case 2 in our series — a 10-year-old girl with M. abscessus cervical lymphadenitis — was initially misdiagnosed with typical bacterial (non-mycobacterial) lymphadenitis, with a diagnostic delay of more than two months. NTM cervical lymphadenitis is characteristically insidious in presentation, with clinical features that substantially overlap with bacterial lymphadenitis; it fails to respond to conventional antibiotic therapy, and the diagnostic sensitivity of acid-fast staining and mycobacterial culture is only approximately 50%–60% (13), contributing to a high rate of missed or delayed diagnosis. In pediatric patients presenting with subacute or chronic cervicofacial masses unresponsive to standard antimicrobial therapy, NTM should be included in the differential diagnosis regardless of immune status, and tissue specimens for microbiological evaluation should be obtained at the earliest opportunity.

Notably, the age distribution and microbiological profile observed in our series diverge from established patterns. Literature (14, 15), primarily reflecting the epidemiological trends in Western populations, suggests that NTM lymphadenitis typically manifests in toddlers (1–5 years) and is most commonly caused by *Mycobacterium avium complex*. However, our cases (Cases 1 and 2) involved older children (9–10 years) infected with *Mycobacterium abscessus*. This discrepancy highlights the unique regional epidemiology in Asia, where rapidly growing mycobacteria, particularly *M. abscessus*, are more prevalent than in Western cohorts. The higher incidence of *M. abscessus* in our region may contribute to the atypical clinical presentation and warrants a more cautious diagnostic approach including tNGS.

## Congenital airway structural anomalies and structural lung disease

Congenital airway structural anomalies — specifically laryngomalacia and bronchomalacia — seem to represent another important category of predisposing factors identified in this series, with their core pathogenic mechanism residing in the sustained impairment of mechanical airway defenses (16, 17). In healthy individuals, resistance to environmental NTM is conferred by coordinated transport transport, adequate expectoration capacity, and the phagocytic and bactericidal functions of alveolar macrophages — even non-activated alveolar macrophages are sufficient to clear conventional infectious inocula from the respiratory tract (18). In contrast, dynamic airway collapse induced by congenital airway malacia results in airflow obstruction, diminished mucociliary clearance, and chronic accumulation of airway secretions, creating a stable ecological niche that facilitates persistent NTM colonization (17). Recurrent airway inflammation further compromises the epithelial barrier, perpetuating a vicious cycle of “structural anomaly → impaired clearance → infectious colonization” (19).

Cystic fibrosis (CF) constitutes a well-established structural lung disease predisposing to NTM infection. In CF patients, CFTR dysfunction results in markedly viscous airway mucus, severely impaired mucociliary clearance, and chronic accumulation of secretions, collectively creating a microenvironment conducive to pathogen colonization (20). Recurrent chronic airway inflammation further damages bronchial epithelium, accelerating the progression of bronchiectasis, which is an independent structural risk factor for NTM infection (20, 21). Notably, there exists a bidirectional, mutually reinforcing relationship between NTM infection and bronchiectasis: sustained infection and inflammation drive bronchiectatic remodeling, while pre-existing bronchiectasis in turn amplifies the risk of NTM acquisition (20, 21). Among pediatric CF patients, *M. abscessus* is the predominant NTM pathogen. A prospective French cohort study enrolling 298 CF patients with a mean age of 11.3 years reported an *M. abscessus* isolation rate of 5%, with a mean age at first isolation of only 11.9 years; notably, 86.7% of infected children had concomitant bronchiectasis (22). Data from the UK Cystic Fibrosis Registry further demonstrated that the NTM culture-positivity rate among CF patients aged ≤16 years rose from 1.3% in 2010 to 3.8% in 2015 — a nearly threefold increase within five years — with the *M. abscessus* complex identified as the predominant pathogen, underscoring that NTM infection in pediatric CF has emerged as a clinically significant and increasingly prevalent challenge (23).

## Systemic immunodeficiency: diGeorge syndrome

In contrast to localized infection, disseminated NTM infection predominantly affects hosts with systemic cellular immunodeficiency. Principal risk factors include: severe HIV/AIDS-associated immunosuppression; iatrogenic immunosuppression following solid organ or hematopoietic stem cell transplantation; inborn errors of immunity involving the NF-*κ*B signaling pathway, GATA2 mutations (as seen in MonoMAC syndrome), neutralizing anti-IFN-*γ* autoantibodies, and genetic defects associated with Mendelian susceptibility to mycobacterial disease; and pharmacological immunosuppression (24–28).

Thymic hypoplasia secondary to DiGeorge syndrome represents one of the most important causes of congenital T-cell immunodeficiency in children (29). The thymus is the central organ for T-cell maturation and selection; thymic aplasia or hypoplasia leads to a profound reduction or near-complete absence of peripheral naïve T cells, rendering affected patients highly susceptible to intracellular pathogens, including NTM (30–32). Hicks et al. reported three infants with complete DiGeorge anomaly who developed disseminated NTM infection during infancy; pathogenic species included the *Mycobacterium avium* complex and *Mycobacterium kansasii*, with dissemination involving the lungs, mediastinal lymph nodes, liver, bone marrow, and skeletal system, further confirming that severe T-cell immunodeficiency resulting from thymic aplasia constitutes a major risk factor for disseminated mycobacterial infection (32).

The DiGeorge syndrome patient in this series was only 6 months of age and presented with congenital laryngomalacia, bronchomalacia, patent ductus arteriosus, patent foramen ovale, and severe malnutrition. His NTM infection can be attributed to the convergent effects of two distinct pathogenic mechanisms: systemic immunodeficiency — manifested as impaired T-cell-mediated intracellular defense secondary to malnutrition and thymic hypoplasia — and congenital airway structural defects, wherein airway malacia caused dynamic collapse and mucociliary dysfunction, providing the structural prerequisite for local *M. abscessus* colonization. When multiple predisposing factors coexist, their pathogenic contributions are not merely additive but synergistically amplified, resulting in more severe infection and substantially greater therapeutic complexity.

## *Mycobacterium paragordonae*: recognition of a rare species

*Mycobacterium paragordonae* was first isolated and described from a pulmonary specimen of a Korean patient in 2014; although phylogenetically closely related to *Mycobacterium gordonae*, it was established as a distinct species on the basis of significant differences in DNA homology (9, 33). Clinical reports of *M. paragordonae* infection remain exceedingly limited, with documented sites of infection including the lungs, spine, and peritoneal cavity, all reported as sporadic cases (9). From an anatomical perspective, the presence of intraparotid lymph nodes renders the parotid gland a predilection site for NTM lymphadenitis (14). The parotid *M. paragordonae* infection in our 9-year-old immunocompetent patient constitutes a rare pediatric NTM case; it was ultimately confirmed by tissue tNGS — underscoring the indispensable diagnostic value of tNGS in the identification of rare mycobacterial species. Although the pathogenicity of *M. paragordonae* has not been fully established, consistent histopathological findings (chronic granulomatous inflammation with focal necrosis and positive acid-fast staining), combined with the patient's favorable clinical response to targeted anti-NTM therapy, support its role as a genuine pathogen rather than a colonizer or contaminant. The patient achieved marked clinical improvement following surgical excision combined with a multidrug anti-NTM regimen, consistent with the available literature (14, 34): for pediatric parotid NTM lymphadenitis, surgical resection represents an effective therapeutic option, either as a primary intervention for diagnostic confirmation or as adjunctive therapy in cases of incomplete response to pharmacological treatment alone, although the risk of facial nerve injury warrants careful preoperative evaluation.

## Diagnostic value of tNGS and therapeutic strategies

tNGS employing multiplex targeted amplicon-based high-throughput sequencing across predefined pathogen panels played a pivotal diagnostic role in this case series. Compared with untargeted metagenomic sequencing, tNGS concentrates sequencing on predefined pathogen targets, rendering detection largely unaffected by host genomic background while enabling species-level identification at reduced cost (35). Compared with conventional culture and acid-fast staining, tNGS offers substantial advantages in the identification of rare mycobacterial species — exemplified by the identification of *M. paragordonae* in Case 1 — evaluation of complex infectious presentations in immunocompromised hosts, and facilitation of early, targeted therapeutic decision-making. It must be emphasized, however, that tNGS results require careful interpretation within the appropriate clinical context. In this series, all tNGS-positive findings were supported by corresponding clinical symptoms, radiological abnormalities, and/or histopathological evidence, and sequence read counts were explicitly documented for each identification, providing transparency in result reporting and effectively mitigating the risk of over-interpretation or misclassification of colonization as infection.

Regarding treatment, all five patients received individualized multidrug anti-NTM regimens tailored to pathogenic species, site of infection, and host immune status, and all achieved clinical improvement prior to discharge. While observation or simple excision might be considered for MAC lymphadenitis in some Western cohorts (15), the higher prevalence of rapidly growing mycobacteria in Asia—particularly *M. abscessus*—may necessitate a more robust combination of surgical debridement and targeted antimicrobial therapy to ensure definitive cure. Notably, for the localized extrapulmonary infections (Cases 1 and 2), surgical intervention was initially performed as a necessary diagnostic measure to obtain tissue for histopathological and microbiological identification in the setting of clinical uncertainty. Following the combination of surgical intervention and targeted antimicrobial therapy, both patients achieved complete clinical resolution with no evidence of recurrence during follow-up. Given the intrinsic resistance of *M. abscessus* conventional antimicrobial agents, therapeutic options are largely limited to amikacin, cefoxitin, imipenem-cilastatin, linezolid, and azithromycin (10, 36); these agents constituted the backbone of the treatment regimens for the *M. abscessus* pulmonary disease cases in this series. In Case 5, the addition of intravenous immunoglobulin and T*α*1 exemplified the integrated therapeutic philosophy of combining targeted antimicrobial therapy with immunomodulatory intervention in the immunocompromised host.

## Summary and limitations

Taken together, whether predisposed by disruption of physical barriers, impaired mechanical airway defense in the setting of structural lung disease, or systemic cellular immunodeficiency, all pathogenic mechanisms ultimately converge on the attenuation of a specific component of host defense — providing environmental NTM an opportunity to transition from colonization to invasive infection. For children with any of the aforementioned predisposing conditions, particularly those presenting with protracted pulmonary infection unresponsive to standard antimicrobial therapy, progressive cervicofacial masses, or unexplained recurrent fever, NTM should be actively incorporated into the differential diagnosis, and tNGS should be considered as an early diagnostic tool when conventional methods are insufficient. Treatment regimens for pediatric NTM infection should be individualized on the basis of species identification, site of infection, antimicrobial susceptibility testing results, and host immune status, with vigilant monitoring for adverse effects associated with prolonged therapeutic courses.

This study has several limitations. The sample size is small, and the follow-up duration is relatively limited, and long-term outcomes warrant further observation. Nevertheless, as a representative illustration of the full predisposing factor spectrum of pediatric NTM infection, the clinical implications of this case series remain informative and are not diminished by the limited sample size. Future multicenter, large-scale studies are needed to further elucidate the epidemiological characteristics of pediatric NTM infection, the relative contributions of individual risk factors, optimal therapeutic strategies, and key determinants of prognosis. Furthermore, tNGS testing in this series was performed selectively using different certified assay panels at multiple accredited laboratories, reflecting the real-world clinical setting of this retrospective case series. The inherent heterogeneity of platforms and analytical pipelines precludes direct inter-case comparison of diagnostic performance metrics such as sensitivity, sequencing depth, or limit of detection. Future prospective studies should ideally employ a standardized tNGS platform and unified bioinformatics pipeline to facilitate robust cross-study comparisons of diagnostic accuracy.

## Data Availability

The original contributions presented in the study are included in the article/Supplementary Material, further inquiries can be directed to the corresponding author.
